# Assessing *Plasmodium falciparum* transmission in mosquito-feeding assays using quantitative PCR

**DOI:** 10.1186/s12936-018-2382-6

**Published:** 2018-07-05

**Authors:** Claire Y. T. Wang, James S. McCarthy, Will J. Stone, Teun Bousema, Katharine A. Collins, Seweryn Bialasiewicz

**Affiliations:** 1 Child Health Research Centre, The University of Queensland, Brisbane, Australia; 2 Centre for Children’s Health Research, Children’s Health Queensland, Brisbane, Australia; 3 QIMR Berghofer Medical Research Institute, Brisbane, Australia; 4 Radboud Institute for Health Sciences, Radboud University Medical Centre, Nijmegen, The Netherlands

**Keywords:** Malaria, *Plasmodium falciparum*, PCR, Transmission, Transmission-blocking, Droplet digital PCR, qPCR, Oocyst, Taqman

## Abstract

**Background:**

Evaluating the efficacy of transmission-blocking interventions relies on mosquito-feeding assays, with transmission typically assessed by microscopic identification of oocysts in mosquito midguts; however, microscopy has limited throughput, sensitivity and specificity. Where low prevalence and intensity mosquito infections occur, as observed during controlled human malaria infection studies or natural transmission, a reliable method for detection and quantification of low-level midgut infection is required. Here, a semi-automated, Taqman quantitative PCR (qPCR) assay sufficiently sensitive to detect a single-oocyst midgut infection is described.

**Results:**

Extraction of genomic DNA from *Anopheles stephensi* midguts using a semi-automated extraction process was shown to have equivalent extraction efficiency to manual DNA extraction. An 18S *Plasmodium falciparum* qPCR assay was adapted for quantitative detection of *P. falciparum* midgut oocyst infection using synthetic DNA standards. The assay was validated for sensitivity and specificity, and the limit of detection was 0.7 genomes/µL (95% CI 0.4–1.6 genomes/µL). All microscopy-confirmed oocyst infected midgut samples were detected by qPCR, including all single-oocyst positive midguts. The genome number per oocyst was assessed 8–9 days after feeding assay using both qPCR and droplet digital PCR and was 3722 (IQR: 2951–5453) and 3490 (IQR: 2720–4182), respectively.

**Conclusions:**

This semi-automated qPCR method enables accurate detection of low-level *P. falciparum* oocyst infections in mosquito midguts, and may improve the sensitivity, specificity and throughput of assays used to evaluate candidate transmission-blocking interventions.

**Electronic supplementary material:**

The online version of this article (10.1186/s12936-018-2382-6) contains supplementary material, which is available to authorized users.

## Background

Despite recent progress with malaria control, it remains a significant global health problem, with an estimated 212 million new cases of malaria occurring in 2015 [1]. Progress to date has been achieved by increasing access to effective anti-malarial treatment and reducing transmission using vector control measures [2]; however, the efficacy of these interventions is threatened by development of drug [3–5] and insecticide [6, 7] resistance. To maintain progress and achieve elimination, existing malaria control measures may need to be supplemented with novel tools. Drugs and vaccines that specifically aim to prevent transmission through mosquito vectors may be particularly effective in reducing malaria incidence and the onward transmission of resistant malaria parasites [8].

The efficacy of transmission-blocking drugs and vaccines is normally evaluated in mosquito-feeding assays that allow mosquitoes to feed on either in vitro cultured gametocytes [9, 10], or gametocytaemic blood from naturally or experimentally infected volunteers [11, 12]. Transmission of malaria to mosquitoes is then assessed by detection of either oocysts within the mosquito midgut or sporozoites in the salivary glands, typically using microscopy. Although microscopy is the gold-standard method for detecting mosquito infection, it has a number of limitations. The technique is labour-intensive and therefore difficult to scale up for large studies [13], it is technically demanding, and it can be difficult to distinguish oocysts from other artefacts—particularly with low-level infections [14]—making it difficult to accurately confirm or exclude single-oocyst infection.

In contrast to the highly infected mosquitoes generated in laboratory mosquito-feeding assays, the intensity of mosquito infection (midgut oocyst numbers) is typically low in mosquito feeding experiments on naturally or experimentally infected subjects, due to the low gametocyte densities present in the mosquitoes blood meal [15–18]. To accurately evaluate the in vivo transmission of malaria in such settings, a large number of mosquitoes must be used in feeding assays, and thus, a high-throughput method capable of sensitive and specific detection of midgut-oocyst infection is needed [19, 20].

Methods have previously been developed to overcome the limitations of microscopy and include ELISA to detect the circumsporozoite protein in mosquito lysates (CSP-ELISA) [21–23], bioluminescence assays to detect transgenic parasites expressing GFP or firefly luciferase [13, 24, 25], and molecular detection of *Plasmodium* DNA [23, 26, 27]. While CSP-ELISA has been reported to be robust and cost effective, variations in sensitivity and specificity have been reported and at present these assays are not truly quantitative [23, 28, 29]. Bioluminescence assays have been particularly useful for increasing the throughput of experiments where transgenic parasites can be used; but these assays cannot be used to evaluate transmission in natural infections or during controlled human malaria infection (CHMI) studies involving wild type parasites. Various PCR-based methods have been successfully used for detection of mosquito infection in different settings [23, 26, 27]; however, while these assays offer increased sensitivity of detection, they are either not fully quantitative, or rely on signal detection by non-specific SYBR-green fluorescent dyes. Taqman hydrolysis probes offer an alternative to SYBR-based real-time PCR, and superior specificity and accuracy due to the additional requirement of probe homology to the specific target in order to generate a positive signal [30, 31]. Taqman hydrolysis probes have been widely adopted for quantitative PCR (qPCR) assays targeting the *Plasmodium* 18S ribosomal RNA gene (hereafter referred to as 18S qPCR) to monitor the development of blood-stage infection during CHMI studies [16, 18, 31–33]. Such assays are routinely able to quantify parasitaemia at levels ~ 100 fold lower than expert thick-film microscopy [34–36]. In this study, an existing 18S Taqman qPCR assay was adapted to assess the prevalence of *P. falciparum* oocyst infection in mosquito midguts. Synthetic plasmid DNA standards were developed and validated to allow quantification of parasite genomes in oocyst-infected midguts, assay controls were used to ensure reproducibility of assay performance, and the assay was adapted to a semi-automated process to allow increased throughput. In addition, the ability of the assay to detect microscopy-confirmed single-oocyst positive midguts was evaluated, and quantification of genome copies by 18S qPCR was compared with absolute quantification by droplet digital PCR (ddPCR) [37] to ensure confidence in estimating midgut parasite burden.

## Methods

### Mosquito rearing

*Anopheles stephensi* mosquitoes (Sind-Kasur Nijmegen strain [38]) were reared at 27 °C and ~ 70–80% relative humidity (RH) on a 12 h day/night cycle and were fed on 8% sucrose with para-aminobenzoic acid (PABA). Mosquitoes between 3 and 5 days post emergence were used for negative controls or were fed on gametocytaemic blood.

### Preparation of mosquito midgut samples for assay validation

For assay optimization negative control midguts were collected from non-blood fed *An. stephensi* mosquitoes, added to lysis buffer as detailed below, and stored at − 20 °C until DNA extraction (n = 124). Twenty-three infected midguts from *An. stephensi* mosquitoes (Radboud University Medical Centre) were used as positive control material. Midguts were collected from mosquitoes 7 days after feeding and were prepared as previously described [23]. They were stained with 1% mercurochrome, examined by microscopy, and oocyst numbers in each midgut recorded. After examination by microscopy, slides were flooded with PBS to allow easy removal of coverslips, and excess mercurochrome was removed by dragging the midgut gently through a clean PBS droplet. Midguts were stored in 40 µL PBS at − 80 °C.

### Preparation of mosquito midgut samples from a CHMI study

Mosquitoes were fed on gametocytaemic blood from volunteers enrolled in a CHMI transmission study via either direct skin feeding or membrane feeding assays [18]. Mosquitoes were dissected for evaluation of oocysts on day 8 or 9 after feeding assay. Midguts were collected in 180 µL of DNA Tissue Lysis buffer (Roche Diagnostics, Australia) for PCR analysis, or were stained with 0.5% mercurochrome for visualization of oocysts by microscopy prior to collection in lysis buffer and PCR analysis.

### Preparation of mosquito midguts for non-oocyst parasite DNA detection

Two concentrations of parasite infected blood meal were prepared from a mixed parasite culture (predominantly gametocytes): 1 gametocyte/µL, which is the average gametocyte density in volunteers during a CHMI transmission study, and 1000 gametocytes/µL, which is greater than the peak parasitaemia in CHMI transmission study volunteers. These parasite-infected blood-meals were heat-inactivated for 20 min at 42 °C prior to feeding to ensure parasites were no longer viable, and therefore unable to develop into oocysts [39, 40]. Two batches of *An. stephensi* mosquitoes were fed with the two different concentrations of parasites, and samples of mosquitoes (n = 7–10) were dissected from each batch at time points between day 1 and day 10 post-infection. Midguts were stored in 180 µL Roche lysis buffer at − 80 °C until DNA extraction.

### Manual DNA extraction

Mosquito midguts were stored in 300 µL ATL buffer (QIAGEN, Australia), spiked with a known amount of whole Equine Herpesvirus (EHV) and stored at − 20 °C until DNA extraction. Prior to extraction, samples were homogenized with acid-washed glass beads (425–600 μm/Cat# G4649-500G, Sigma-Aldrich) on the Tissue Lyser (QIAGEN, Australia) at 30 oscillation/s for 2 min. Supernatant was collected and 40 µL of proteinase K was added and the sample incubated at 56 °C overnight. Nucleic acid extraction was performed the next day using the DNeasy Tissue & Blood kit (QIAGEN, Australia) following the manufacturer’s protocol. Total nucleic acid was eluted into 50 µL and stored at − 80 °C until PCR was performed.

### Semi-automated DNA extraction

Mosquito midguts were stored in 180 µL DNA Tissue Lysis buffer (Roche Diagnostics, Australia) spiked with a known amount of EHV. 20 µL of Proteinase K was added to the lysis mixture, followed by incubation overnight at 56 °C. Total nucleic acid was extracted from the midgut lysates using the DNA and Viral NA Small Volume Kit on the MagNA Pure 96 instrument (Roche Diagnostics, Australia) following the manufacturer’s protocol (DNATissue SV2.0). Total nucleic acid was eluted into 100 µL and stored at − 80 °C until PCR was performed.

### RPS7 and EHV PCR assays for extraction quality control

A Taqman hydrolysis probe PCR assay targeting the ribosomal RNA protein S7 (RPS7) gene [41] of *An. stephensi* (Genbank Accession No. AF539918) was designed (Table 1). In silico analysis of the PCR primers and probes using BLASTn (NCBI) showed high specificity to *An. stephensi,* with some sequence homology to ortholog genes in several other mosquito species (e.g.: *Anopheles gambiae*, *Aedes aegypti*). No predicted primer interactions leading to PCR amplification in non-mosquito templates were observed. The RPS7 assay was analysed on a 1:10 dilution series of whole mosquito DNA extracts, with the expected logarithmic progression of dilution series (from Cp 19 to 35) being observed (see Additional file 1).

**Table 1:** Primer and probe sequences used in this study

Oligonucleotide names	Sequence	Target	References
PerFal	5′-CTTTTGAGAGGTTTTGTTACTTTGAGTAA-3′	*P. falciparum* 18S rRNA gene	[31]
	5′-TATTCCATGCTGTAGTATTCAAACACA-3′		
	5-FAM-TGTTCATAACAGACGGGTAGTCATGATTGAGTTCA-BHQ1′		
RPS7	5′-TGGAAATGAACTCGGATCTGAAG-3′	*An. stephensi* rRNA protein S7 gene (RPS7)	[41]
	5′-CCTTCTTGTTGTTGAACTCGACCT-3′		This study
	5′-HEX-CAGCTGCGTGATCTGTACATCACCCGCGC BHQ1′		This study
EHV	5′-GATGACACTAGCGACTTCGA-3′	Equine Herpesvirus	[42]
	5′-CAGGGCAGAAACCATAGACA-3′		
	5′-QUASAR670 -TTTCGCGTGCCTCCTCCAG-BHQ1′		

A second PCR assay (Table 1) targeting the spiked EHV was run in conjunction with the RPS7 assay to monitor extraction efficiency and inhibition of PCR [42]. The QuantiNova Probe PCR Kit (Qiagen, Australia) was used for all midgut testing. Each 10 µL PCR reaction consisted of 0.4 µM of each primer, 0.2 µM of probe, Rox reference dye (1:200) and 4 µL of template DNA. Amplification was performed on a ViiA7 Real-Time PCR System (Life Technologies, Australia) in a 384-well format, with the following cycling conditions: 95 °C heat activation for 2 min, 45 fast cycles of 95 °C for 5 s and 60 °C for 5 s. Sample quality and DNA extraction efficiency was considered acceptable if the EHV and RPS7 PCR crossing point (Cp) values fell within 2 standard deviations (SD) of the mean Cp within each extraction/PCR run. These limits of acceptability (mean Cp ± 2 SD) were based on recommendations by the minimum information for publication of quantitative real-time PCR experiments (MIQE) guidelines and Burd [43, 44] to account for variations within 95% confidence interval for evaluating qPCR assay variances.

For comparison of DNA extraction methods total nucleic acid was extracted from uninfected midguts using the manual method (n = 62) or the semi-automated method (n = 62) and analysed using the RPS7 and EHV PCR assays. Cp values for both PCR assays obtained from the manual extraction method were adjusted (1 Cp) based on DNA template doubling at each PCR cycle [45] to reflect the 1:2 difference in elution volume before data analysis.

## 18S qPCR assay

*Plasmodium falciparum* parasite quantification was undertaken using a previously published Taqman qPCR assay targeting the 18S rRNA gene (Table 1) [31]. In silico analysis of 18S qPCR oligonucleotide specificity using the NCBI and PlasmoDB [46] databases showed 100% identity to the *P. falciparum* 3D7 18S rRNA gene, and no predicted cross-reactivity with any other human *Plasmodium* species. Further characterization of the assay was performed based on the MIQE guidelines [44], including limit of detection (LOD) using synthetic plasmid dilution series, as well as specificity and repeatability (intra-assay variability). The synthetic DNA standards (syn18S DNA) consisted of the 18S qPCR target cloned into a pMA-T plasmid backbone (Thermo Fisher Scientific, Australia) and serially diluted in uninfected whole blood nucleic acid extract to give final concentrations of 7.2 × 10^4^ to 0.1 DNA copies/µL. Six replicates of each standard were analysed with the 18S qPCR assay three times on separate days to determine the assay analytical sensitivity and repeatability [43]. Six uninfected human whole blood extracts were included as negative controls in each qPCR run. Additional uninfected blood extracts (n = 54) from volunteers were analysed on 18S qPCR assay to determine assay specificity.

Quantification of 18S rDNA copies in midguts was achieved using a standard curve generated from the syn18S DNA dilutions. Copy numbers were converted to genome numbers based on the alignment of qPCR assay to NCBI reference *P. falciparum* 3D7 genome in Geneious software (Geneious version 10.2, Biomatters [47]) and the number of PCR assay binding sites per genome was determined to be 3.

## 18S ddPCR

The 18S qPCR assay was adapted to the droplet digital PCR format. Triplicate syn18S DNA serial dilutions and 14 microscopy-confirmed oocyst-positive midguts (oocyst number: 1–3) from a CHMI transmission study were extracted on the MagNA Pure 96 and analysed in duplicate on the QX-200 ddPCR system (BioRad, Australia) to obtain absolute quantification of parasite genomes. The ddPCR reactions were prepared using ddPCR Supermix for Probes (no dUTP) following manufacturer’s protocol. Each 20 µL ddPCR reaction contained 0.9 µM of each 18S primer, 0.25 µM of Taqman probe, 1 µL of HAEIII restriction enzyme mix (5 U/µL) (Promega, Australia), and 5 µL of DNA template. Amplification was performed on a C1000 Touch thermal cycler (BioRad, Australia) following the conditions of 95 °C for 10 min, 40 cycles of 94 °C for 30 s and 60 °C for 60 s, with final step of 98 °C for 10 min and 4 °C forever. Genome quantification was determined using the QuantaSoft analysis software (Bio-Rad, Australia). Reactions containing uninfected human whole blood or midgut nucleic acid extracts were used to determine the negative amplitude threshold for the synthetic controls and parasite positive midguts, respectively. Droplet count per sample ranged from 17,547 to 21,044, with a mean fluorescence amplitude of 7816 for positive and 702 for negative samples (see Additional file 2).

### Statistical analysis

Statistical analysis was performed using GraphPad Prism (ver. 7.03) or STATA (version 14.2, StataCorp, College Station, Texas). The relative extraction efficiencies of the manual and automated methods were compared using the Cp values generated from EHV and RPS7 PCR assays and Student’s *t* test to determine if two methods produced comparable nucleic acid yields. 18S qPCR assay LOD was estimated by probit analysis and defined as the concentration at which 95% of the samples test positive (SPSS ver. 25, SPSS, Inc, Chicago, IL).

## Results

### Validation of 18S qPCR for genome quantification

To quantify the number of genomes detected in the mosquito midguts using the 18S qPCR assay, a standard curve was generated using synthetic plasmid DNA. From three independent PCR reactions assay efficiency was consistently greater than 90% (range 90–99%). The LOD_95%_ was determined to be 2.1 DNA copies/µL (95% CI 1.3–4.7 copies/µL) which translates to 0.7 genomes/µL. False-positives were not observed in the negative controls of the three PCR runs (see Additional file 3) or the additional 54 uninfected human blood extracts, demonstrating assay specificity of 100%. Precision was assessed using the Cp values of amplification curves from each dilution to derive standard deviations (SD) and % CV. To ensure a robust estimation of precision, Cp values greater than 40 (n = 2; Cp 41.2—1.4 copies/µL and Cp 43 0.7 copies/µL) were excluded from the analysis. The mean SD and  % CV from the three PCR runs was 0.69 (pooled SD across standards) and 2.07 respectively, showing good repeatability (see Additional file 3).

### Evaluation of midgut DNA extraction methods

The RPS7 PCR assay was developed to evaluate the efficiency of DNA extraction from midgut tissue. Preliminary evaluation using DNA extracts from whole mosquitoes (n = 5) or midguts (n = 15), resulted in amplification of the appropriate size PCR product, with subsequent DNA sequence analysis confirming the correct amplicon identity. Mean Cp values for whole mosquitoes and isolated midguts were 19.2 and 23.6, respectively (midgut Cp range 22–27, due to variation in midgut tissue size) (see Additional file 4). No amplification was observed in uninfected human whole blood (n = 12), or in nucleic acid extracted from cultured *P. falciparum*-infected RBCs (n = 12) demonstrating specificity.

The RPS7 and EHV PCR assays [42] were used to evaluate the efficiency of manual DNA extraction using midguts from mosquitoes fed on cultured *P. falciparum* gametocytes and collected in EHV-spiked buffer (n = 96). All extracts were positive in both PCR assays with average Cp values of 24.75 (95% CI 24.68–24.82) for EHV, and 22.85 (95% CI 22.68–23.02) for RPS7 (Table 2). The EHV Cp values from the midgut extracts were equivalent to those obtained from EHV-spiked PBS (Cp 24–25) indicating no PCR inhibition in midgut tissue extracts. RPS7 Cp values showed higher variability due to the varied size of the mosquitoes and their midguts, but the variation was small (CV < 5%). *Plasmodium* DNA could also be detected using this extraction method with 100% agreement between 18S qPCR and microscopy results. Eleven microscopy-confirmed *P. falciparum* oocyst-positive midguts (oocyst number ranging 1–4) were evaluated; all had EHV and RPS7 Cp values within the acceptable QC range (mean Cp ± 2 SD [43]) (Table 2) and all had *P. falciparum* DNA detected by 18S qPCR.

**Table 2:** Manual extraction efficiency evaluated using EHV and RPS7 markers

PCR target	Midguts from mosquitoes fed on gametocyte culture Mean Cp (95% CI)	Midguts with microscopy-confirmed *P. falciparum* oocysts Mean Cp (95% CI)
EHV	24.75 (24.68–24.82)	24.14 (23.68–24.61)
RPS7	22.85 (22.68–23.02)	23.03 (22.0–24.07)

Mean Cp and 95% CI values displayed for the EHV and RPS7 PCR analysis of *An. stephensi* mosquito midguts fed on gametocyte culture with unknown infection status (n = 96) or with microscopy-confirmed *P. falciparum* oocysts (n = 11/oocyst numbers range 1–4). The 11 microscopy-positive midguts were also confirmed positive by 18S qPCR

**Fig. 1:**
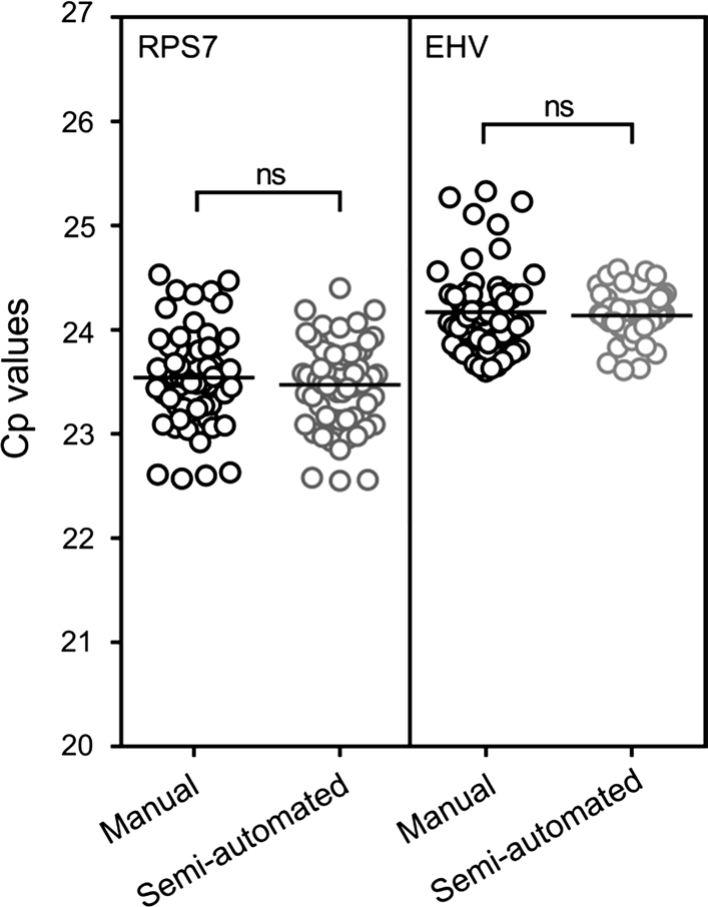
Comparison of DNA extraction efficiency using manual and semi-automated processes. DNA was extracted from two batches of 62 midguts using either the manual or semi-automated methods with efficiency compared by measuring the RPS7 and EHV DNA. The lines indicate the group mean and the groups were compared by Student’s *t* test (*p *= 0.38 and *p* = 0.56 for RPS7 and EHV, respectively)

To enable large scale processing (> 2000 midguts) during CHMI transmission studies, a semi-automated DNA extraction method was evaluated and efficiency was compared to manual method using the RPS7 and EHV PCR assays. Cp values obtained for the two PCR assays were not significantly different (EHV *p* = 0.56 and RPS7 *p* = 0.38) comparing the two DNA extraction methods by Student’s *t* test (Fig. 1). Therefore, the semi-automated method showed equivalent performance to the manual method and is suitable for large scale processing.

The semi-automated extraction method also allowed the detection of low-intensity midgut oocyst infections. 12 microscopy-confirmed oocyst-positive midguts (oocyst number: 1–9) were evaluated and all were positive by 18S qPCR assay, including detection of all single-oocyst positive midguts (n = 7; see Additional file 5).

Further evaluation using 2344 midguts collected during a CHMI transmission study [18] demonstrated that the RPS7 PCR assay can be used to monitor midgut collection and DNA extraction quality during large scale processing. Genomic DNA was extracted using the semi-automated method and the RPS7 mean Cp was 23.16 (95% CI 23.13–23.19). Using the acceptability criteria for the RPS7 assay of mean Cp ± 2 SD, 1.5% (35/2344) of the midguts fell outside the QC range suggesting partial loss of tissue or incomplete tissue lysis.

### Specificity of the 18S qPCR assay for oocyst detection

18S qPCR performed on midguts 7–10 days after mosquito feeding assay was shown to be specific for developing oocysts, and residual asexual parasite or gametocyte DNA from the blood-meal was not detected. This was determined by evaluating midguts from mosquitoes fed on heat-inactivated gametocytes (1 or 1000 gametocytes/µL). On day 1 post-feeding, *P. falciparum* DNA was detected in all mosquito midguts (Table 3) with average 18S Cp values of 38.5 and 33.1 for mosquitoes fed on the 1 and 1000 gametocytes/µL culture, respectively. By day 2, 18S qPCR was negative in all mosquitoes fed on the 1 gametocyte/µL blood meal and in 9/10 mosquitoes (90%) fed on the 1000 gametocytes/μL blood meal. The 18S qPCR was negative in all mosquitoes evaluated between day 3 and 10 indicating residual non-oocyst *P. falciparum* DNA is not detected from 3 days after mosquito feeding.

**Table 3:** Detection of non-oocyst *P. falciparum* DNA in mosquito midguts after a blood meal

Days post-infection	1 parasite/µL blood meal (18S positive/total)	1000 parasites/µL blood meal (18S positive/total)
D1	10/10 (mean Cp 38.5; SD = 2.4)	9/9 (mean Cp 33.1; SD = 1.0)
D2	0/7	1/10 (Cp 38.4)
D3	0/10	0/10
D6	0/10	0/10
D7	0/10	0/10
D8	0/10	0/10
D9	0/10	0/10
D10	0/10	0/10

### Midgut genome quantification using ddPCR and qPCR

**Fig. 2:**
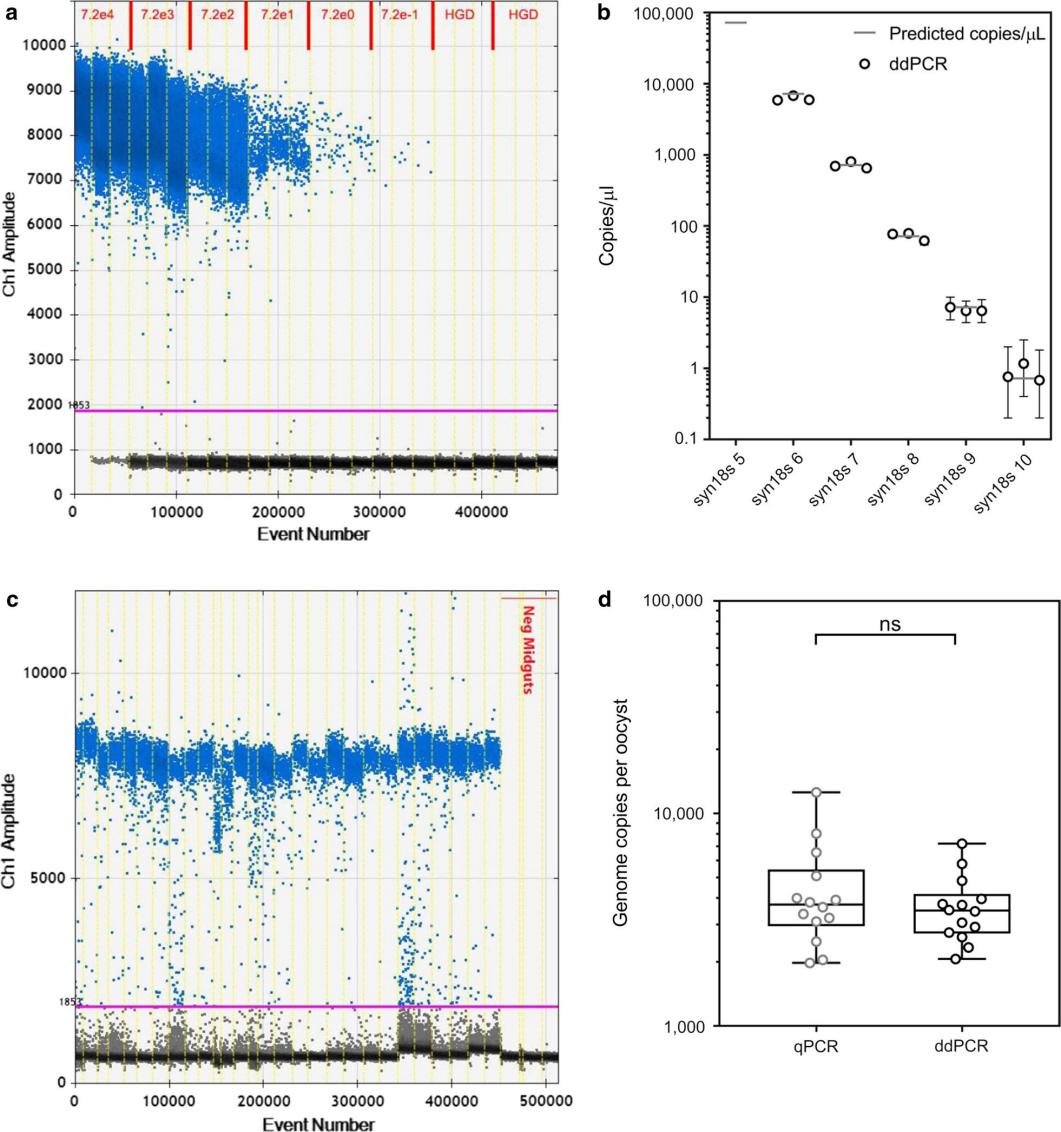
Droplet digital PCR for oocyst genome quantification. **a** One-dimensional scatter plots showing 18S ddPCR assay on positive (syn18s standards from 7.2 × 10^4^ to 0.72 copies/µL) and negative human blood (“HGD”) extracts. Clear demarcation between positive and negative partitions is shown. Uninfected human blood extract reaction was used to determine a universal positive/negative threshold set at 1853. **b** Quantification of syn18s standards using ddPCR in triplicate (black circles indicate the median of each run with error bars showing 95% CI) compared to the predicted qPCR value (grey line). **c** One-dimensional scatter plots showing 18S ddPCR assay on 14 oocyst-positive midguts, with positive and negative partitions. Uninfected mosquito midguts (“Neg”) were used to determine a universal positive/negative threshold set at 1853. **d** Quantification of genomes per oocyst for the14 microscopy-confirmed oocyst-positive midguts using 18S qPCR and 18S ddPCR. Box plots indicate the median and whiskers show the minimum and maximum responses. Groups compared using Wilcoxon matched-pairs signed rank test (*p* = 0.43)

High concordance between ddPCR and qPCR quantification was observed when using both syn18S DNA plasmid standards and microscopy-confirmed oocyst-positive midguts from a CHMI transmission study [18]. A universal positive/negative threshold was set at a fluorescence amplitude of 1853 using uninfected human blood nucleic acid extracts or uninfected midguts (Fig. 2a + c). Using the syn18S DNA plasmid standards, the ddPCR assay was unable to accurately quantify target copy numbers above 7.2 × 10^3^ DNA copies/µL due to the reaction not generating sufficient numbers of negative droplets to perform the Poisson distribution calculations (i.e.: the saturation point of the assay) (Fig. 2a). All other syn18S DNA standards including the lowest dilution (0.72  DNA copies/µL) were detected by ddPCR, with quantification of copy numbers closely matching the predicted qPCR syn18S DNA copy numbers (Fig. 2b and Table 4).

**Table 4:** 18S ddPCR with synthetic DNA standards

	qPCR		ddPCR (DNA copies/µL)	
	qPCR Cp^a^	Predicted DNA copies/µL^b^	Total^c^	Individual mean^c^ (95% CI^d^)
syn18s-5	19.5	72,100	Saturated	Saturated (n/a)
syn18s-6	22.7	7210	6176	6198.7 (6790–5600)
syn18s-7	26.1	721	714	717.3 (804–628)
syn18s-8	29.4	72	72	72.4 (83.2–61.6)
syn18s-9	33.3	7.2	6.6	6.7 (8.1–8.3)
syn18s-10	36.7	0.72	0.84	0.9 (1.5–0.4)

^a^ qPCR Cp values for the synthetic standards are shown as means (n = 18). No quantification results produced for qPCR as the synthetic standards were used to produce standard curve

^b^ syn18s DNA predicted copies/µL as calculated based on Qubit reading

^c^ Triplicate ddPCR reaction data for each dilution is shown as total and individual amounts, where the total amount was calculated based on the triplicate reaction data being treated as one larger sample

^d^ ddPCR 95% confidence intervals (CI) are shown based on the total data calculations

ddPCR using midguts produced more variable fluorescence values with less clear demarcation between positive and negative droplets compared to ddPCR using the syn18S templates (Fig. 2c). However, the 18S ddPCR assay was able to detect parasite genomes in all microscopy-confirmed oocyst-positive midguts (oocyst numbers ranging from 1 to 3). There was no difference in the number of genomes per oocyst calculated using ddPCR compared to qPCR (*p* = 0.43; Fig. 2d, see Additional file 6), with the median genome number per oocyst estimated to be 3490 (IQR: 2720–4182) by ddPCR and 3722 (IQR: 2951–5453) by qPCR.

## Discussion

This study describes the development and validation of a higher throughput PCR based method for accurately detecting low-level midgut oocyst infections with no false positives. The assay is sufficiently sensitive to detect midgut infection with just a single oocyst, and it does not detect *P. falciparum* DNA in mosquito midguts from 3 days after a blood meal is taken unless oocyst infection has been established. Furthermore, oocyst parasite genomes can be quantified, and the values obtained by qPCR were validated by absolute quantification with droplet digital PCR.

Current methods to evaluate efficacy of transmission to mosquitoes include microscopy, CSP-ELISA, bioluminescence assays and PCR. However, each method suffers limitations including a lack of assay sensitivity and specificity, low-throughput, non-quantitative outputs [22, 24, 28, 48, 49] or the requirement for use of transgenic parasites [24]. Here a semi-automated qPCR-based method with a Taqman hydrolysis probe was used for increased assay specificity and sensitivity, to allow objective quantification of parasite genomes, and to enable increased throughput for large-scale transmission studies.

To use PCR for evaluating transmission to mosquitoes, appropriate control assays are required to ensure variability in sample quality, extraction efficiency, or PCR inhibition do not occur [43]. In this study, two quality control PCR assays were used, one to monitor DNA extraction efficiency (EHV) and the other to monitor midgut collection and extraction quality (RPS7). The *Anopheles* RPS7 assay was specifically designed to ensure the presence of midgut tissue without partial sample loss, and to confirm complete lysis of the tissue during extraction. Variation is expected in the Cp values obtained using the RPS7 assay due to the varying size of the midgut tissue in the mosquitoes being evaluated. Analysis of 2344 midguts from a CHMI study enabled us to assess the expected range in midgut tissue size and resulting RPS7 Cp values, and ensure natural variation falls within our QC acceptability range (mean Cp ± 2 SD), and values falling outside the QC range would indicate a failure in tissue sampling or processing. These QC assays used a relative QC acceptability range (mean Cp ± 2SD) instead of an absolute Cp value range, allowing the protocol to be easily adapted to new settings where the mosquito colony, age, size, or rearing protocols may vary causing shifts in the QC indicators. These quality control assays were also used to compare the efficiency of DNA extraction using manual and semi-automated methods. The semi-automated extraction method resulted in an equivalent yield of PCR target to the manual extraction approach, but with considerably increased throughput.

The 18S qPCR assay used in this study has been previously described [31] and validated against FACS quantified cultured parasites in whole blood. To quantify genomes within oocysts, synthetic DNA standards were generated using a plasmid containing a single copy of the assay target. The analytical sensitivity of the assay was determined according to stringent guidelines [43, 44], resulting in a LOD of 2.1 DNA copies/µL (or 0.7 genomes/µL). Using the synthetic DNA standards, the 18S qPCR assay was able to quantify parasite genomes in all known oocyst-positive samples. Single-oocyst positive midguts were consistency detected across multiple extraction methods and PCR platforms, thereby demonstrating the assay’s potential utility in settings where low-prevalence and low-intensity infections are expected, such as CHMI transmission studies [18] and natural transmission [17, 50]. Residual non-oocyst *P. falciparum* DNA did not persist for more than 2 days after the mosquito blood meal and, therefore, does not interfere with oocyst detection, in accordance with previous observations [27].

Limited information exists on the impact of mercurochrome on DNA recovery and its use in PCR. This study, in agreement with a previous report [51], demonstrates the capacity for successful retrieval and detection of parasite DNA from mercurochrome-stained midguts. However, the impact of mercurochrome, a potential intercalating agent [52], on DNA quality and integrity was not specifically assessed in these studies and may require further investigation.

Quantification of genome numbers per oocyst were equivalent using the 18S qPCR and 18S ddPCR assays with median genomes per oocyst being 3722 and 3490, respectively. Two recent studies using ddPCR for detection of malaria in culture and *Plasmodium* infected subjects also reported good agreement in parasite estimates between ddPCR and qPCR, but with improved sensitivity and precision observed using ddPCR, particularly in low-density infections [53, 54]. An additional advantage of ddPCR is the ability to accurately quantify parasites without the need for external standards, making it easier to compare data between laboratories [53]. However, despite these potential benefits, the close agreement between the two PCR technologies found in this study shows that qPCR can still be used in field studies and other situations where ddPCR instruments are not available.

In the samples evaluated here, genome numbers per oocyst were consistent irrespective of oocyst number per midgut suggesting that quantification of genome numbers could be used to enumerate oocysts. However, only low intensity infections (oocyst number ≤ 3) were assayed, thus further evaluation with a greater range of oocyst numbers would be required to fully investigate the relationship between parasite genomes and oocyst numbers. It is possible that this relationship may be non-linear, and with higher infection intensity (more oocysts) lower numbers of genomes may be present per oocyst [24]. This qPCR assay may therefore allow for a more accurate estimation of mosquito infectivity by quantifying numbers of infectious sporozoites instead of numbers of oocysts, which may be more relevant when assessing the potential for onward transmission [55]. Although the PCR based assay developed here is sensitive, provides robust and accurate estimation of parasite infections in mosquitoes, and has increased throughput due to the use of semi-automated DNA extraction, at present it still uses dissected midguts. Dissection of the mosquitoes limits a further increase in throughput, but it may be possible to adapt this method to allow processing of whole mosquitoes [23], or pools of whole mosquitoes and to assess salivary gland sporozoite infection intensity. The RPS7 and EHV control assays will facilitate the future development and evaluation of such assays.

## Conclusions

A sensitive method for detecting oocyst-specific *P. falciparum* DNA in mosquito midguts was successfully developed with capacity for increased specificity and throughput. QC criteria were established for sample processing and applied to quantify low-level oocyst infections in mosquito midguts. The emergent ddPCR technology was used to verify results and demonstrate its utility in future CHMI studies. This assay shows promise as a tool to evaluate transmission-blocking interventions both in experimental CHMI transmission studies and in malaria-endemic settings.

## Additional files

**Additional file 1.** The logarithmic progression of RPS7 PCR assay on mosquito DNA extract serial dilutions. The RPS7 PCR assay was further analysed on serially diluted mosquito DNA extracts to demonstrate the assay’s logarithmic progression (Cp range from 18 to 35). The PCR assay has 100% efficiency across the 6 dilutions with slope of -3.28 and a coefficient of correlation (R^2^) of 0.998.

**Additional file 2.** 18S ddPCR absolute quantification data output of triplicate synthetic control serial dilutions. 18S ddPCR data output of triplicate synthetic control serial dilutions, including calculated copies/µl of extract. Reactions were saturated at the fifth (syn18S-5) dilution, and therefore did not provide sufficient data for Poisson distribution calculations.

**Additional file 3.** 18S qPCR validation data using syn18S DNA standard dilution series performed on 3 separate occasions. HGD – human genomic DNA (uninfected human blood extracts) as negative controls. ND = not detected. Average linear regression from 3 PCR runs Cp=-3.4541*log(conc)+36.1006.

**Additional file 4.** Cp values obtained using the RPS7 PCR assay on DNA extracts from whole *A. stephensi* mosquitoes and mosquito midguts. The preliminary evaluation of RPS7 PCR assay successfully amplified DNA extracted from 5 whole *A. stephensi* mosquitoes and 15 mosquito midguts, producing mean Cp of 19.2 for whole mosquito DNA extract Cp of 23.6 for midgut extracts.

**Additional file 5.** Detection of low intensity midgut infections. EHV, RPS7, and 18S Cp values for 12 *P. falciparum* positive and two negative mosquito midgut controls, as well as numbers of oocysts identified by microscopy. Acceptable QC range was defined as within 2 SD of the mean Cp. *Inhibited extraction or PCR. Midgut 1-6, 8-13 were positively infected. Midgut 7 & 14 were uninfected midguts.

**Additional file 6.** 18S ddPCR results compared with qPCR in microscopy confirmed oocyst-positive midguts. Comparison of qPCR and ddPCR quantification of oocyst-positive midguts from a CHMI study. The median genome number per oocysts were estimated to be 3,722 genomes/oocyst by qPCR and 3,490 genomes/oocyst by ddPCR.

## Abbreviations

CHMI: controlled human malaria infection
qPCR: quantitative polymerase chain reaction
ddPCR: droplet digital PCR
NA: nucleic acid
LOD: limit of detection
SD: standard deviation
CV: coefficient of variation
RPS7: ribosomal RNA protein S7
EHV: equine herpes virus
MIQE: minimum information for publication of quantitative real-time PCR experiments
