# Supplementary material for: Assessing Plasmodium falciparum transmission in mosquito-feeding assays using quantitative PCR
Source: Malar J. 2018 Jul 5;17:249. doi: 10.1186/s12936-018-2382-6 (PMC6034226; doi:10.1186/s12936-018-2382-6)
Supplement: Supplementary file 1 — Additional file 1. The logarithmic progression of RPS7 PCR assay on mosquito DNA extract serial dilutions. The RPS7 PCR assay was further analysed on serially diluted mosquito DNA extracts to demonstrate the assay’s logarithmic progression (Cp range from 18 to 35). The PCR assay has 100% efficiency across the 6 dilutions with slope of -3.28 and a coefficient of correlation (R2) of 0.998. [file 12936_2018_2382_MOESM1_ESM.docx]

### Additional files

**Additional file 1. The logarithmic progression of RPS7 PCR assay on mosquito DNA extract serial dilutions.**

| **sample ID** | **Cp** |
| --- | --- |
| A. stephensi whole mosquito extract neat | 18.25 |
| A. stephensi whole mosquito extract 1:10 | 21.06 |
| A. stephensi whole mosquito extract 1:100 | 24.47 |
| A. stephensi whole mosquito extract 1:1000 | 27.62 |
| A. stephensi whole mosquito extract 1:10000 | 30.82 |
| A. stephensi whole mosquito extract 1:100000 | 34.7 |
| uninfected human blood DNA extract (n=2) | ND |

The RPS7 PCR assay was further analysed on serially diluted mosquito DNA extracts to demonstrate the assay’s logarithmic progression (Cp range from 18 to 35). The PCR assay has 100% efficiency across the 6 dilutions with slope of -3.28 and a coefficient of correlation (R^2^) of 0.998.
