# Supplementary material for: Assessing Plasmodium falciparum transmission in mosquito-feeding assays using quantitative PCR
Source: Malar J. 2018 Jul 5;17:249. doi: 10.1186/s12936-018-2382-6 (PMC6034226; doi:10.1186/s12936-018-2382-6)
Supplement: Supplementary file 2 — Additional file 2. 18S ddPCR absolute quantification data output of triplicate synthetic control serial dilutions. 18S ddPCR data output of triplicate synthetic control serial dilutions, including calculated copies/µl of extract. Reactions were saturated at the fifth (syn18S-5) dilution, and therefore did not provide sufficient data for Poisson distribution calculations. [file 12936_2018_2382_MOESM2_ESM.docx]

**Additional file 2. 18S ddPCR absolute quantification data output of triplicate synthetic control serial dilutions.**

| **Sample** | **Predicted copies/µl** | **Copies/µl of extract** | **95% CI Max (copies/µl extract)** | **95% CI Min (copies/µl extract)** | **Positives** | **Negatives** | **Accepted Droplets** | **Threshold** | **Mean Amplitude of Positives** | **Mean Amplitude of Negatives** | **Mean Amplitude Total** |
| --- | --- | --- | --- | --- | --- | --- | --- | --- | --- | --- | --- |
| syn18S-5 | 72100 | 4,000,000 | 4,000,000.0 | 40,000.0 | 17547 | 0 | 17547 | 1853 | 8590.9 | 0 | 8590.9 |
| syn18S-5 | 72100 | 28,680 | 30,200.0 | 27,320.0 | 18121 | 41 | 18162 | 1853 | 8217.4 | 748.75 | 8200.5 |
| syn18S-5 | 72100 | 27,720 | 29,040.0 | 26,480.0 | 18734 | 52 | 18786 | 1853 | 8216.4 | 764.1 | 8195.8 |
| syn18S-6 | 7210 | 5,872 | 5,980.0 | 5,768.0 | 13234 | 5331 | 18565 | 1853 | 7993.2 | 716.27 | 5903.6 |
| syn18S-6 | 7210 | 6,788 | 6,908.0 | 6,668.0 | 14406 | 4461 | 18867 | 1853 | 8200.5 | 739.41 | 6436.3 |
| syn18S-6 | 7210 | 5,936 | 6,040.0 | 5,832.0 | 14174 | 5604 | 19778 | 1853 | 7618.2 | 710.04 | 5660.8 |
| syn18s -7 | 721 | 696 | 724.0 | 672.0 | 2751 | 17250 | 20001 | 1853 | 7831.8 | 692.73 | 1674.7 |
| syn18s -7 | 721 | 804 | 832.0 | 776.0 | 2987 | 16047 | 19034 | 1853 | 7616 | 696.48 | 1782.4 |
| syn18s -7 | 721 | 652 | 676.0 | 628.0 | 2719 | 18325 | 21044 | 1853 | 7441.2 | 689.19 | 1561.6 |
| syn18s -8 | 72 | 77 | 85.2 | 68.4 | 315 | 19180 | 19495 | 1853 | 7713.5 | 685.41 | 798.97 |
| syn18s -8 | 72 | 79 | 87.2 | 70.4 | 340 | 20096 | 20436 | 1853 | 7825.8 | 693.98 | 812.64 |
| syn18s -8 | 72 | 62 | 68.8 | 54.0 | 273 | 20761 | 21034 | 1853 | 7692.3 | 696.08 | 786.89 |
| syn18s -9 | 7.2 | 7.20 | 10.0 | 4.8 | 30 | 19725 | 19755 | 1853 | 7955.5 | 690.59 | 701.62 |
| syn18s -9 | 7.2 | 6.40 | 8.8 | 4.4 | 28 | 20996 | 21024 | 1853 | 7660.9 | 682.82 | 692.11 |
| syn18s -9 | 7.2 | 6.40 | 9.2 | 4.4 | 28 | 20403 | 20431 | 1853 | 7778.7 | 702.08 | 711.77 |
| syn18s -10 | 0.72 | 0.76 | 2.0 | 0.2 | 3 | 19015 | 19018 | 1853 | 7321.6 | 689.18 | 690.22 |
| syn18s -10 | 0.72 | 1.16 | 2.5 | 0.4 | 5 | 20527 | 20532 | 1853 | 7562.2 | 698.75 | 700.42 |
| syn18s -10 | 0.72 | 0.68 | 1.8 | 0.2 | 3 | 20938 | 20941 | 1853 | 7458.6 | 692.14 | 693.11 |
| Human Whole Blood Extract | - | 0 | 0.7 | 0.0 | 0 | 19599 | 19599 | 1853 | 0 | 688.29 | 688.29 |
| Human Whole Blood Extract | - | 0 | 0.7 | 0.0 | 0 | 20748 | 20748 | 1853 | 0 | 687.66 | 687.66 |
| Human Whole Blood Extract | - | 0 | 0.8 | 0.0 | 0 | 18647 | 18647 | 1853 | 0 | 671.23 | 671.23 |
|  |  |  |  |  |  |  |  | mean | 7816 | 702 |  |

18S ddPCR data output of triplicate synthetic control serial dilutions, including calculated copies/µl of extract. Reactions were saturated at the fifth (syn18S-5) dilution, and therefore did not provide sufficient data for Poisson distribution calculations
