# Supplementary material for: Assessing Plasmodium falciparum transmission in mosquito-feeding assays using quantitative PCR
Source: Malar J. 2018 Jul 5;17:249. doi: 10.1186/s12936-018-2382-6 (PMC6034226; doi:10.1186/s12936-018-2382-6)
Supplement: Supplementary file 3 — Additional file 3. 18S qPCR validation data using syn18S DNA standard dilution series performed on 3 separate occasions. HGD – human genomic DNA (uninfected human blood extracts) as negative controls. ND = not detected. Average linear regression from 3 PCR runs Cp=-3.4541*log(conc)+36.1006. [file 12936_2018_2382_MOESM3_ESM.docx]

**Additional file 3. 18S qPCR validation data using syn18S DNA standard dilution series performed on 3 separate occasions.**

| **syn18S DNA (copies/µL)/(replicates)** | **mean Cp (SD)** | **Cp range**  **(min, max)** | **% 18S qPCR positive** | **Cp-CV (%)** |
| --- | --- | --- | --- | --- |
| 7.21 ×10^4^(18) | 19.49 (0.19) | 19.17, 19.83 | 18 (100%) | 0.95 |
| 7.21 ×10^3^(18) | 22.73 (0.42) | 21.16, 23 | 18 (100%) | 1.85 |
| 7.21 ×10^2^ (18) | 26.13 (0.35) | 25.07, 26.62 | 18 (100%) | 1.35 |
| 7.21 ×10^1^ (18) | 29.39 (0.53) | 28.09, 30.16 | 18 (100%) | 1.82 |
| 14.4 (18) | 32.04 (0.54) | 30.65, 32.84 | 18 (100%) | 1.69 |
| 7.2 (18) | 33.25 (0.71) | 31.72, 34.59 | 18 (100%) | 2.15 |
| 3.6 (18) | 34.04 (0.98) | 32.04, 35.78 | 18 (100%) | 2.88 |
| 1.4 (18) | 35.7 (0.84) | 33.92, 41.23 | 15 (83%) | 2.36 |
| 0.7 (18) | 36.24 (1.30) | 34.3, 43.01 | 13 (72%) | 3.59 |
| 0.1 (18) | 36.66 | n/a | 1 (6%) | n/a |
| HGD (18) | ND | n/a | 0% | n/a |
|  | Mean SD (pooled) 0.69 |  |  | Mean CV  2.6 |

HGD – human genomic DNA (uninfected human blood extracts) as negative controls. ND = not detected. Average linear regression from 3 PCR runs Cp=-3.4541*log(conc)+36.1006.
