# Supplementary material for: Assessing Plasmodium falciparum transmission in mosquito-feeding assays using quantitative PCR
Source: Malar J. 2018 Jul 5;17:249. doi: 10.1186/s12936-018-2382-6 (PMC6034226; doi:10.1186/s12936-018-2382-6)
Supplement: Supplementary file 4 — Additional file 4. Cp values obtained using the RPS7 PCR assay on DNA extracts from whole A. stephensi mosquitoes and mosquito midguts. The preliminary evaluation of RPS7 PCR assay successfully amplified DNA extracted from 5 whole A. stephensi mosquitoes and 15 mosquito midguts, producing mean Cp of 19.2 for whole mosquito DNA extract Cp of 23.6 for midgut extracts. [file 12936_2018_2382_MOESM4_ESM.docx]

**Additional file 4. Cp values obtained using the RPS7 PCR assay on DNA extracts from whole *A. stephensi* mosquitoes and mosquito midguts**

| **sample ID** | **Cp** | **mean Cp** |
| --- | --- | --- |
| A stephensi whole mosquito extract neat -1 | 18.25 | 19.2 |
| A stephensi whole mosquito extract neat -2 | 18.9 |  |
| A stephensi whole mosquito extract neat -3 | 19.28 |  |
| A stephensi whole mosquito extract neat -4 | 19.48 |  |
| A stephensi whole mosquito extract neat -5 | 19.87 |  |
| midgut extract -1 | 22.11 | 23.6 |
| midgut extract -2 | 23.57 |  |
| midgut extract -3 | 25.03 |  |
| midgut extract -4 | 27.02 |  |
| midgut extract -5 | 24.88 |  |
| midgut extract -6 | 25.02 |  |
| midgut extract -7 | 25.5 |  |
| midgut extract -8 | 23.46 |  |
| midgut extract -9 | 22.52 |  |
| midgut extract -10 | 22.12 |  |
| midgut extract -11 | 22.78 |  |
| midgut extract -12 | 22.07 |  |
| midgut extract -13 | 23.19 |  |
| midgut extract -14 | 22.44 |  |
| midgut extract -15 | 22.44 |  |
| Uninfected human whole blood extracts (n=12) | ND | n/a |
| Malaria culture serial dilutions (3.19x10^6^ to 3.19p/ml; n=12) | ND |  |

The preliminary evaluation of RPS7 PCR assay successfully amplified DNA extracted from 5 whole *A. stephensi* mosquitoes and 15 mosquito midguts, producing mean Cp of 19.2 for whole mosquito DNA extract Cp of 23.6 for midgut extracts.
