# Supplementary material for: Assessing Plasmodium falciparum transmission in mosquito-feeding assays using quantitative PCR
Source: Malar J. 2018 Jul 5;17:249. doi: 10.1186/s12936-018-2382-6 (PMC6034226; doi:10.1186/s12936-018-2382-6)
Supplement: Supplementary file 5 — Additional file 5. Detection of low intensity midgut infections. EHV, RPS7, and 18S Cp values for 12 P. falciparum positive and two negative mosquito midgut controls, as well as numbers of oocysts identified by microscopy. Acceptable QC range was defined as within 2 SD of the mean Cp. *Inhibited extraction or PCR. Midgut 1-6, 8-13 were positively infected. Midgut 7 & 14 were uninfected midguts. [file 12936_2018_2382_MOESM5_ESM.docx]

**Additional file 5. Detection of low intensity midgut infections**

| **SAMPLE ID** | **EHV Cp** | **RPS7 Cp** | **18s Cp** | **Oocyst number** |
| --- | --- | --- | --- | --- |
| midgut 1 | 24.7 | 23.5 | 29.3 | 2 |
| midgut 2 | 25.4 | 24.8 | 33.4 | 1 |
| midgut 3 | 24.9 | 23.9 | 31.6 | 1 |
| midgut 4 | 24.8 | 24.6 | 33.4 | 1 |
| midgut 5 | 24.8 | 22.6 | 27.1 | 1 |
| midgut 6 | 25.0 | 22.8 | 27.3 | 1 |
| midgut 7 (negative) | 24.8 | 22.9 | ND | 0 |
| midgut 8 | 25.2 | 23.0 | 29.1 | 9 |
| midgut 9 | 24.9 | 23.1 | 29.3 | 5 |
| midgut 10 | 26.6* | 25.8* | 33.5* | 2 |
| midgut 11 | 24.5 | 22.7 | 27.2 | 6 |
| midgut 12 | 24.6 | 23.8 | 34.5 | 1 |
| midgut 13 | 25.0 | 23.9 | 31.8 | 1 |
| midgut 14 (negative) | 24.7 | 22.4 | ND | 0 |
| mean Cp | 25.0 | 23.6 | 30.6 |  |
| SD | 0.512 | 0.994 |  |  |
| CV (%) | 2.049 | 4.221 |  |  |
| Acceptable QC range (Mean Cp±2SD) | 23.97-26.01 | 21.57-25.55 |  |  |

EHV, RPS7, and 18S Cp values for 12 *P. falciparum* positive and two negative mosquito midgut controls, as well as numbers of oocysts identified by microscopy. Acceptable QC range was defined as within 2 SD of the mean Cp. *Inhibited extraction or PCR. Midgut 1-6, 8-13 were positively infected. Midgut 7 & 14 were uninfected midguts.
