# Supplementary material for: Assessing Plasmodium falciparum transmission in mosquito-feeding assays using quantitative PCR
Source: Malar J. 2018 Jul 5;17:249. doi: 10.1186/s12936-018-2382-6 (PMC6034226; doi:10.1186/s12936-018-2382-6)
Supplement: Supplementary file 6 — Additional file 6. 18S ddPCR results compared with qPCR in microscopy confirmed oocyst-positive midguts. Comparison of qPCR and ddPCR quantification of oocyst-positive midguts from a CHMI study. The median genome number per oocysts were estimated to be 3,722 genomes/oocyst by qPCR and 3,490 genomes/oocyst by ddPCR. [file 12936_2018_2382_MOESM6_ESM.docx]

**Additional file 6. 18S ddPCR results compared with qPCR in microscopy confirmed oocyst-positive midguts**.

|  |  | **Mean parasite genomes/oocyst** | |
| --- | --- | --- | --- |
| **Midgut ID** | **Oocyst #** | **qPCR** | **ddPCR** |
| 1 | 1 | 2497 | 2753 |
| 2 | 1 | 3917 | 3967 |
| 3 | 1 | 3365 | 3713 |
| 4 | 1 | 3102 | 3067 |
| 5 | 2 | 3821 | 3513 |
| 6 | 2 | 3224 | 3467 |
| 7 | 3 | 5086 | 5800 |
| 8 | 1 | 2047 | 2620 |
| 9 | 1 | 1981 | 2340 |
| 10 | 1 | 12583 | 7220 |
| 11 | 1 | 3623 | 2067 |
| 12 | 2 | 3999 | 2923 |
| 13 | 1 | 8053 | 4827 |
| 14 | 1 | 6554 | 3747 |

Comparison of qPCR and ddPCR quantification of oocyst-positive midguts from a CHMI study. The median genome number per oocysts were estimated to be 3,722 genomes/oocyst by qPCR and 3,490 genomes/oocyst by ddPCR.
